# Current landscape of dyslipidemia-related randomized clinical trials registered on the International Clinical Trials Registry Platform

**DOI:** 10.3389/fpubh.2025.1554858

**Published:** 2025-05-01

**Authors:** Ling Pei, Muzhen Su, Reyihannisha Yakeya, Zhaoqian Hu, Aikedan Abudurexiti, Xiaochan Lin, Hongmei Zhao, Gulisitan Abudourexiti, Abudulimu Sidike, Xiaoli Li

**Affiliations:** ^1^Department of Endocrinology and Metabolism, The First Affiliated Hospital of Jinan University, Guangzhou, China; ^2^Department of Endocrinology, The First People’s Hospital of Kashgar Region, Kashgar, China; ^3^Department of Endocrinology, The First People’s Hospital of Zhaoqing, Guangdong, China; ^4^Department of Emergency, The Third Affiliated Hospital of Guangzhou University of Chinese Medicine, Guangzhou, China

**Keywords:** dyslipidemia, randomized clinical trials, publication, International Clinical Trials Registry Platform, adherence

## Abstract

**Introduction:**

This study elucidates the characteristics of randomized clinical trials (RCTs) related to dyslipidemia that are registered on the International Clinical Trials Registry Platform (ICTRP) to better identify research hotspots, address existing gaps, and improve clinical trial designs.

**Methods:**

This cross-sectional study included dyslipidemia-related RCTs registered on the ICTRP up to 13 August 2024. We evaluated the relevant characteristics of these RCTs and reviewed their publication status after enrollment using PubMed.

**Results:**

A total of 2,410 dyslipidemia-related RCTs were analyzed. The number of registered RCTs sharply increased in 2005 (*N* = 125). The majority of the RCTs included adults (91.4%), with a median sample size of 93 (50–229), and 92.9% of these trials had no sex-based enrollment restrictions. Few RCTs focused on participants aged ≤18 years (2.8%), those aged 19–44 years (3.4%), or exclusively women (2.8%). Medication (83.1%) was the most common type of intervention. Efficacy and safety outcomes were predominant (81.5%), while only 0.7% of the RCTs specified treatment adherence as a primary outcome. The RCTs involving adults had larger sample sizes (median 100.0 [50, 245] vs. 56.0 [27, 108], *p =* 0.047) and lower proportions of natural medicine and extracts (26.6% vs. 33.6%, *p* < 0.001) compared to age-specific RCTs. After enrollment, the 7-year cumulative publication rate was 20.8%.

**Conclusions:**

High-quality RCTs involving younger participants, women, and adherence-related outcomes were lacking. Researchers should prioritize exploring novel therapeutic strategies to improve trial publication rates.

## 1 Introduction

Dyslipidemia is defined as having abnormal levels of total cholesterol (TC), low-density lipoprotein cholesterol (LDL-C), high-density lipoprotein cholesterol (HDL-C), and triglycerides (TG), or any combination of these components. It is a major risk factor for cardiovascular disease (CVD) and stroke (1, 2). In 2008, the global prevalence of elevated plasma TC levels in adults was approximately 39%, and currently, approximately 4 million deaths per year are attributed to LDL-C abnormalities (3, 4). With rapid urbanization and changes in dietary habits and lifestyle, plasma cholesterol levels remain elevated. In addition, the global burden of dyslipidemia has increased over the past 30 years (5). Controlling plasma lipid levels to reduce the risk of CVD and related deaths in the coming decades warrants attention.

Randomized clinical trials (RCTs) are the gold standard for evaluating interventions and guiding clinical decision-making. To ensure standardized registration and information disclosure of clinical research, the International Committee of Medical Journal Editors proposed the registration of trials on a public platform before participant recruitment in 2004 (6). In 2005, the World Health Organization facilitated the establishment of the International Clinical Trials Registry Platform (ICTRP), which collects records of registered RCTs from various registration centers worldwide. The ICTRP integrates data from 18 WHO-endorsed primary registries across various global regions, including ClinicalTrials.gov (United States), the EU Clinical Trials Register (Europe), ChiCTR (China), CTRI (India), PACTR (Africa), ANZCTR (Australia/New Zealand), and registries from Latin America.^1^ Ongoing or completed clinical trials are assigned a unique registration number and standardized data within the ICTRP (7).

Understanding the characteristics of dyslipidemia-related clinical trials can guide improvements in trial design and help identify areas requiring further research. However, systematic evaluations of dyslipidemia-related RCTs are lacking. Therefore, in this study, the characteristics of dyslipidemia-related RCTs registered on the ICTRP were investigated.

## 2 Methods

### 2.1 Searching and selecting relevant registered trials

The ICTRP was searched on 13 August 2024 using the following search strategy: “dyslipidemia” OR “hyperlipidemia” OR “lipid disorders” OR “hypercholesterolemia” OR “hypertriglyceridemia” OR “blood lipid disorders.” Data were downloaded in the form of XML files and transferred into an Excel file to facilitate further data cleaning and analysis. Duplicated trials, observational trials, non-RCTs, and trials that had been withdrawn or had unknown status were excluded. LP and MS systematically removed studies unrelated to dyslipidemia.

### 2.2 Data extraction

The variables were independently extracted by two investigators (LP and MS) after calibrating the extraction criteria. These variables included sex, age, location, sample size, type of registration, status, masking, type of intervention, funding source, intervention mode, and primary outcome. The participants were divided into the following age groups: ≤18, 19 to 44, 45 to 64, and ≥65 years. For further data analysis, these participants were merged into “age-specific groups.” If the age range of the participants spanned two or more subgroups (19–44, 45–64, and ≥65 years), they were classified into the “adult group.” The “adult group” included participants without age restrictions. If an industry was listed as the lead funder, the trial was classified as industry-funded.

### 2.3 Searching publication status of the included trials

Two investigators (RH and ZH) searched PubMed by entering registration numbers in all fields. The search for publication status was updated and finalized by 30 September 2024. Publication of RCTs was confirmed by matching the brief titles and study characteristics outlined on the ICTRP with descriptions in the published articles. The earliest publication date was recorded when multiple publications existed for the same registered trial. Unpublished trials underwent a second publication search by investigators AA and Xiaochan Lin. Any disagreements or uncertainties were resolved through consensus.

### 2.4 Statistical analysis

The number (percentage) of categorical variables and the median (interquartile range) of continuous variables were calculated. The χ^2^ test was performed to compare the categorical variables. Kaplan–Meier analysis was conducted to analyze cumulative publication rates after trial enrollment. All statistical tests were conducted using SPSS version 25.0 (IBM Corporation), and a two-sided *p*-value of <0.05 indicated statistical significance.

## 3 Results

### 3.1 Distribution of dyslipidemia-related RCTs

**Figure 1:**
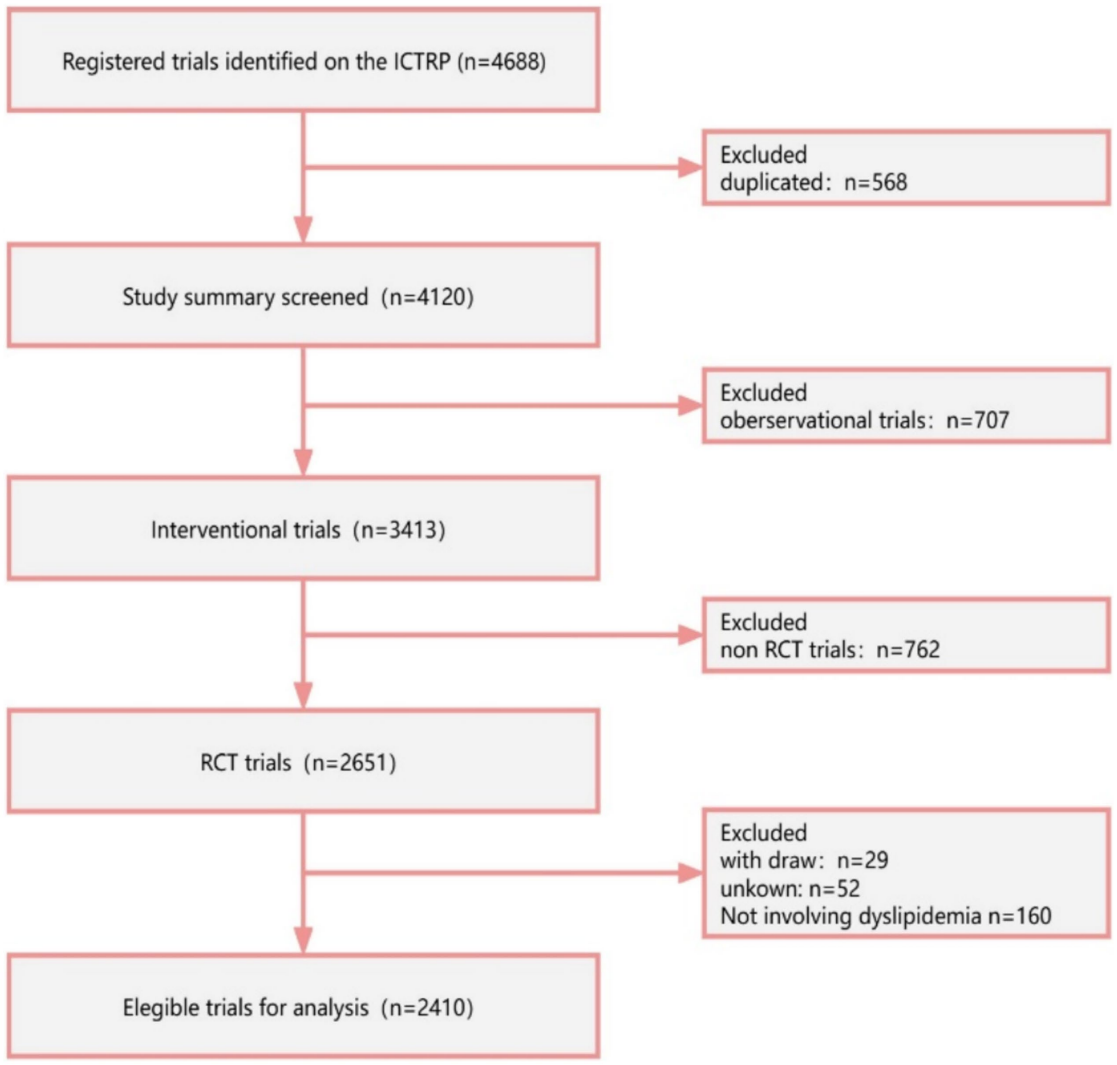
Flowchart of trial selection.

**Figure 2:**
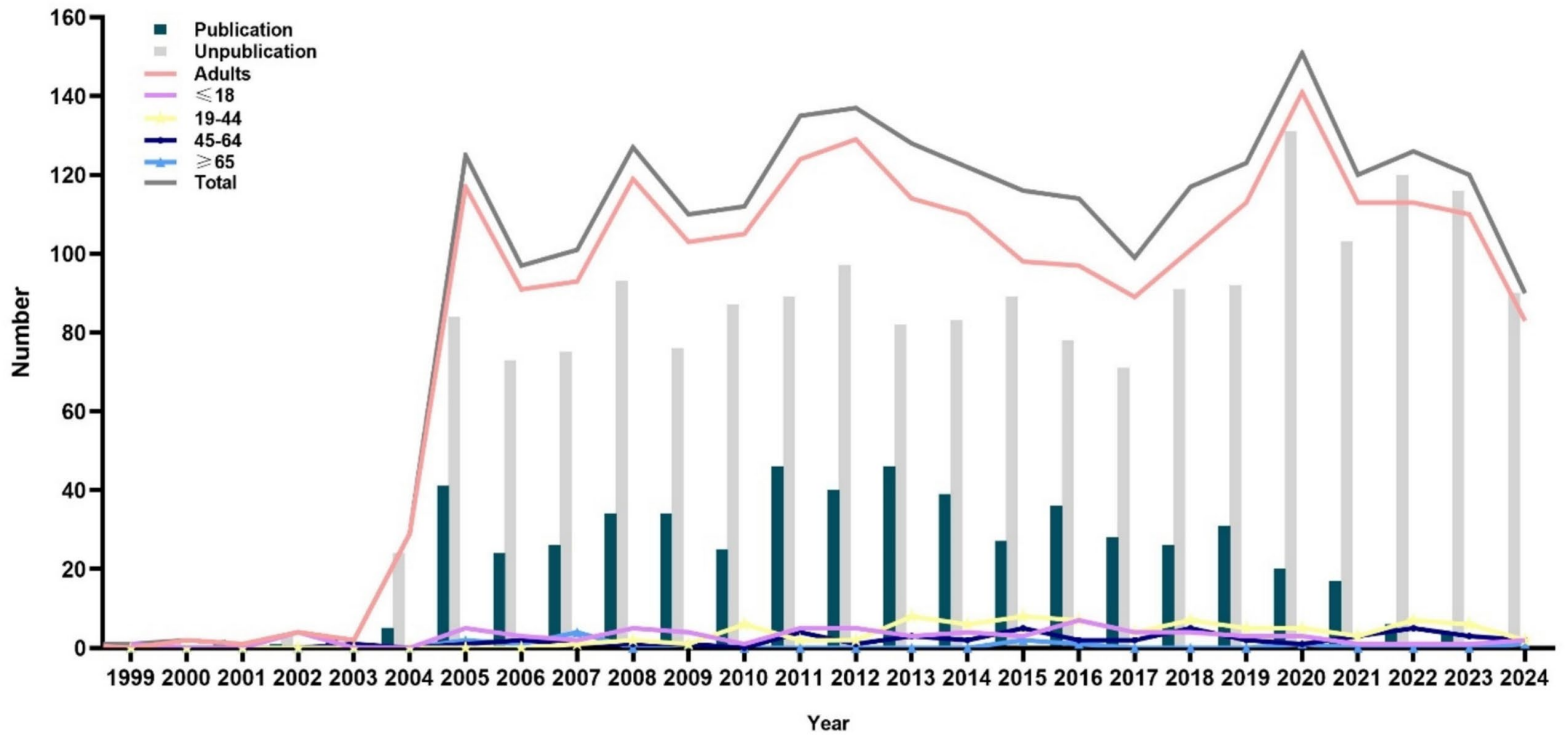
Distribution of dyslipidemia-related randomized clinical trials according to the registered year.

A total of 4,688 registered RCTs were retrieved from the ICTRP. Out of these, 568 duplicated trials, 707 observational trials, and 762 non-RCTs were excluded. Furthermore, trials that were withdrawn (*n* = 29), had unknown status (*n* = 52), and were irrelevant to lipids (*n* = 160) were excluded. Finally, 2,410 RCTs were analyzed (Figure 1). Figure 2 illustrates the distribution of RCTs by registration year from 1997 to 2024. The annual number of registrations increased from 1 in 1997 to 157 in 2020. The number of registered RCTs increased dramatically in 2005 (*n* = 125) (Figure 2).

### 3.2 General characteristics of the included RCTs

Overall, the majority of trials included adults (91.4%), with a median sample size of 93 (50–229) and no sex-based enrollment restrictions (92.9%). Few trials enrolled participants aged ≤18 years (2.8%), those aged 19 to 44 years (3.4%), or exclusively women (2.8%). Approximately half of the trials were registered after patient recruitment, and 64% utilized a blinding method in their design. The majority of the trials had a “not recruiting” status (87.7%). However, only 23.2% of the trials were published. Approximately 42% of the trials were industry-funded. The majority of the trials were conducted in Asia (41.6%), followed by North America (18.0%) and Europe (17.1%). Parallel assignment (78.7%) was the most frequently used intervention model, followed by crossover assignment (14.6%), factorial assignment (1.7%), and sequential assignment (1.2%). Medication (83.1%) was the most common type of intervention. The types of medication included natural medicine and extracts (27.1%), drug combinations (22.9%), statins (10.7%) and emerging lipid-lowering drugs (10.6%), cholesterol absorption inhibitors (2.5%), fibrates (3.1%), niacin (1.3%), omega-3 fatty acids (4.4%), proprotein convertase subtilisin/kexin type 9 (9.3%), and others (7.9%). The primary outcome predominantly focused on efficacy and safety (81.5%). The secondary outcomes included pharmacokinetics (4.9%), plaque and CVD (4.4%), endothelial function and inflammation (3.6%), adherence (0.7%), and others (5.0%) (Table 1).

**Table 1:** Characteristics and study design of all included RCTs (*N* = 2,410).

Item and subcategory	Number (%) or median (IQR)
Sex	
Both female and male subjects	2,240 (92.9%)
Female subjects	68 (2.8%)
Male subjects	102 (4.2%)
Age	
Adults	2,202 (91.4%)
≤18^a^	67 (2.8%)
19–44	82 (3.4%)
45–64	46 (1.9%)
≥65	13 (0.5%)
Location	
Europe	412 (17.1%)
Asia	1,002 (41.6%)
North America	434 (18.0%)
Oceania	43 (1.8%)
South America	48 (2.0%)
Africa	9 (0.4%)
Multi-continent	413 (17.1%)
Sample size	93 (50.0–229.0)
Less than 50	638 (26.5%)
50–99	634 (26.3%)
100–200	440 (18.3%)
More than 200	642 (26.6%)
NA	56 (2.3%)
Type of registration	
Prospective	1,176 (48.8%)
Retrospective	1,234 (51.2%)
Status	
Recruiting	297 (12.3%)
Not recruiting	2,113 (87.7%)
Publication	
Unpublished	1,852 (76.8%)
Published	558 (23.2%)
Masking	
Blinding	1,542 (64.0%)
Open label	593 (24.6%)
NA	275 (11.4%)
Funder	
Industry	1,011 (42.0%)
Non-industry	1,366 (56.7%)
NA	33 (1.4%)
Intervention model	
Parallel assignment	1,896 (78.7%)
Sequential assignment	30 (1.2%)
Factorial assignment	42 (1.7%)
Crossover assignment	352 (14.6%)
NA	90 (3.8%)
Type of intervention	
Medication	2,002 (83.1%)
Management style	82 (3.4%)
Lifestyle	259 (10.7%)
Others	67 (2.8%)
Medication classification^b^	
Statin	215 (10.7%)
Cholesterol absorption inhibitor	51 (2.5%)
Fibrates	62 (3.1%)
Niacin	27 (1.3%)
Omega-3 fatty acids	88 (4.4%)
PCSK9	187 (9.3%)
Emerging lipid-lowering drugs	213 (10.6%)
Natural medicine and extracts	542 (27.1%)
Drug combination	458 (22.9%)
Others	159 (7.9%)
Primary outcome	
Efficacy and safety	1,964 (81.5%)
Pharmacokinetics	117 (4.9%)
Adherence	17 (0.7%)
Plaque and CVD	106 (4.4%)
Endothelial function and inflammation	86 (3.6%)
Others	120 (5.0%)

a Group contains two trials with a maximum inclusion age of 19 years.

b Rounded to one decimal place using standard rounding rules.

IQR, inter-quartile range; NA, not available; RCTs, randomized clinical trials.

### 3.3 Comparison of RCT characteristics based on participants

Table 2 summarizes a comparison of characteristics between the RCTs involving adults and those with age-specific participants. Recruitment status and masking did not differ between the two groups. The RCTs involving adults were more frequently conducted in Asia (43.2% vs. 34.1%), designed with parallel assignment (79.5% vs. 69.7%), multi-centered (18.0% vs. 15.9%), and involved drug combinations (23.7% vs. 12.8%). These RCTs were also more frequently funded by the industry (43.8% vs. 22.6%) and had medication interventions (84.2% vs. 71.2%), compared to the RCTs with age-specific participants (all *p* < 0.001). The trials involving adults had larger sample sizes than those with age-specific adults (median 100.0 [50, 245] vs. 56.0 [27, 108], *p =* 0.047; [sample size >200] 28.4% vs. 7.7%, *p* < 0.001). In addition, approximately half (46.6%) of the trials with age-specific participants had a sample size of <50, compared to only 24.6% of the trials involving adults. However, the proportion of RCTs focusing on natural medicine and extracts (33.6% vs. 26.6%, *p* < 0.001) and those designed with crossover assignment (23.6% vs. 13.8%) was higher in the age-specific group. The trials in the adult group focused more on efficacy and safety (81.9% vs. 76.9%, *p* < 0.016) than those in the age-specific group. Both the adult and age-specific groups paid minimal attention to adherence (1.0% vs. 0.7%).

**Table 2:** Characteristics and study design of randomized controlled trials according to age.

Variables	Adult group (*N* = 2,202)	Age-specific group (*N* = 208)	*p*-value
Sex			<0.001
Both female and male subjects	2,086 (94.7%)	154 (74.0%)	
Female subjects	40 (1.8%)	28 (13.5%)	
Male subjects	76 (3.5%)	26 (12.5%)	
Location			<0.001
Europe	372 (16.9%)	40 (19.2%)	
Asia	952 (43.2%)	71 (34.1%)	
North America	403 (18.3%)	42 (20.2%)	
Oceania	38 (1.7%)	5 (2.4%)	
South America	34 (1.5%)	14 (6.7%)	
Africa	6 (0.3%)	3 (1.4%)	
Multi-continent	397 (18.0%)	33 (15.9%)	
Sample size			
	100 (50.0–245.0)	56 (27.0–108.0)	0.047
Less than 50	541 (24.6%)	97 (46.6%)	<0.001
50–99	584 (26.5%)	50 (24.0%)	
100–200	404 (18.3%)	36 (17.3%)	
More than 200	626 (28.4%)	16 (7.7%)	
NA	47 (2.1%)	9 (4.3%)	
Type of registration			0.002
Prospective	1,096 (49.8%)	80 (38.5%)	
Retrospective	1,106 (50.2%)	128 (61.5%)	
Status			0.719
Recruiting	273 (12.4%)	24 (11.5%)	
Not recruiting	1,929 (87.6%)	184 (88.5%)	
Masking			0.496
Blinding	1,411 (64.1%)	131 (63.0%)	
Open label	536 (24.3%)	57 (27.4%)	
NA	255 (11.6%)	20 (9.6%)	
Funder			<0.001
Industry	964 (43.8%)	47 (22.6%)	
Non-industry	1,207 (54.8%)	159 (76.4%)	
NA	31 (1.4%)	2 (1.0%)	
Intervention model			0.001
Parallel assignment	1,751 (79.5%)	145 (69.7%)	
Sequential assignment	29 (1.3%)	1 (0.5%)	
Factorial assignment	36 (1.6%)	6 (2.9%)	
Crossover assignment	303 (13.8%)	49 (23.6%)	
NA	83 (3.8%)	7 (3.4%)	
Type of intervention			<0.001
Medication	1,853 (84.2%)	149 (71.2%)	
Management style	76 (3.5%)	6 (2.9%)	
Lifestyle	209 (9.5%)	50 (24.0%)	
Others	64 (2.9%)	3 (1.4%)	
Medication classification			<0.001
Statin	197 (10.6%)	18 (12.1%)	
Cholesterol absorption inhibitor	45 (2.4%)	6 (4.0%)	
Fibrates	60 (3.2%)	2 (1.3%)	
Niacin	27 (1.5%)	0	
Omega-3 fatty acids	74 (4.0%)	14 (9.4%)	
PCSK9	173 (9.3%)	14 (9.4%)	
Emerging lipid-lowering drugs	205 (11.1%)	8 (5.4%)	
Natural medicine and extracts	492 (26.6%)	50 (33.6%)	
Drug combination	439 (23.7%)	19 (12.8%)	
Others	141 (7.6%)	18 (12.1%)	
Primary outcome			0.016
Efficacy and safety	1,804 (81.9%)	160 (76.9%)	
Pharmacokinetics	96 (4.4%)	21 (10.1%)	
Adherence	15 (0.7%)	2 (1.0%)	
Plaque and CVD	98 (4.5%)	8 (3.8%)	
Endothelial function and inflammation	79 (3.6%)	7 (3.4%)	
Others	110 (5.0%)	10 (4.8%)	

Data are expressed as number (percentage) or median (inter-quartile range).

NA, not available; RCTs, randomized clinical trials.

**Figure 3:**
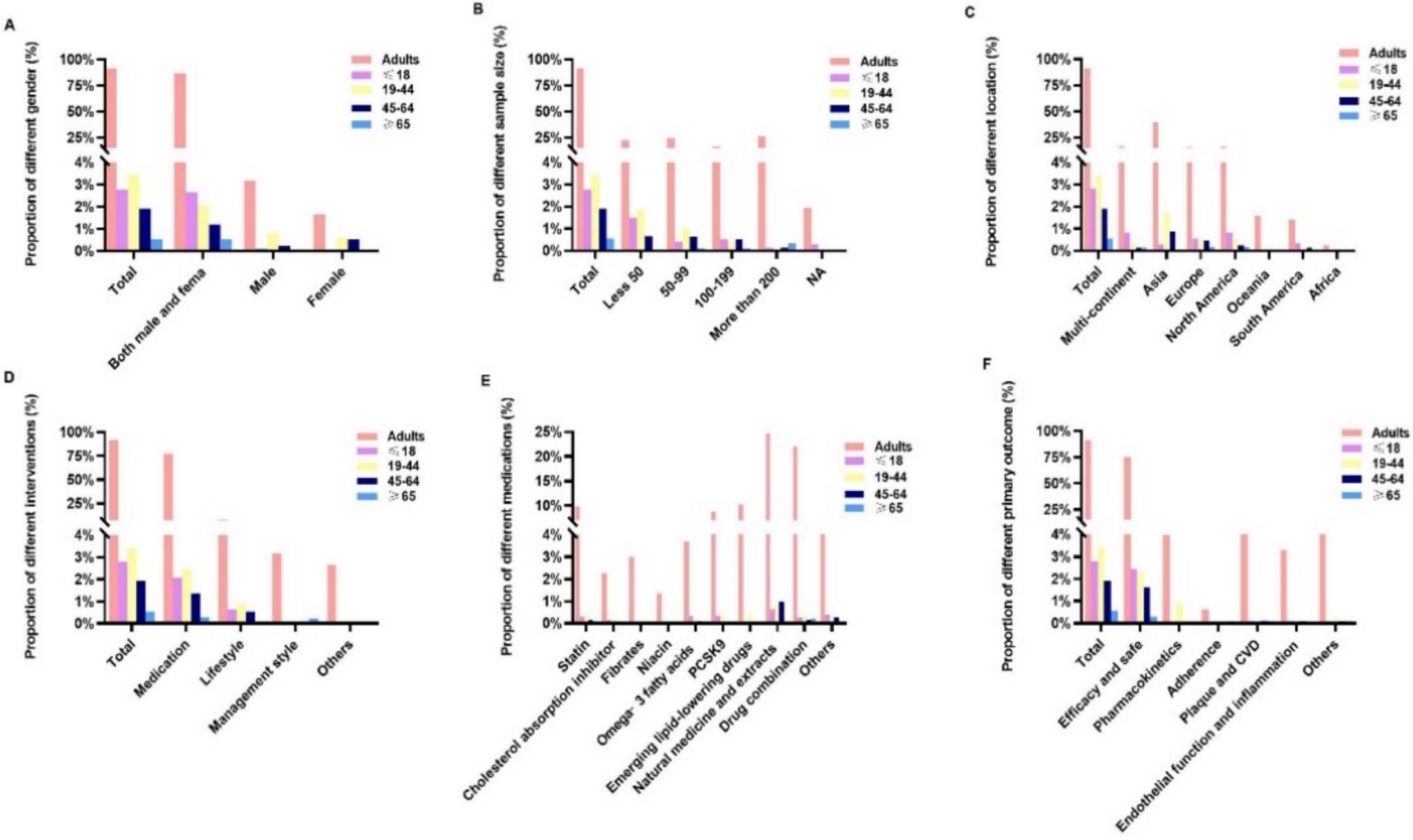
Comparison of the characteristics of dyslipidemia-related randomized clinical trials across different age groups: **(A)** sex, **(B)** sample size, **(C)** location, **(D)** intervention type, **(E)** medication, and **(F)** primary outcomes.

Figure 3 illustrates the distribution of RCTs based on age groups: ≤18, 19 to 44, 45 to 64, and ≥65 years. Only 3.4% of the trials involved participants aged 19 to 44 years, followed by participants aged ≤18 years (2.8%) and 45 to 64 years (1.9%). Only 0.5% of the trials involved participants aged ≥65 years. In addition, only 2.07, 2.45, 1.37, and 0.29% of the RCTs focused on medication for participants aged ≤18, 19 to 44, 45 to 65, and ≥65 years, respectively. Trials were rarely (<1%) conducted on women, adherence, or management interventions within each age-specific group.

### 3.4 Publication status of the RCTs

**Figure 4:**
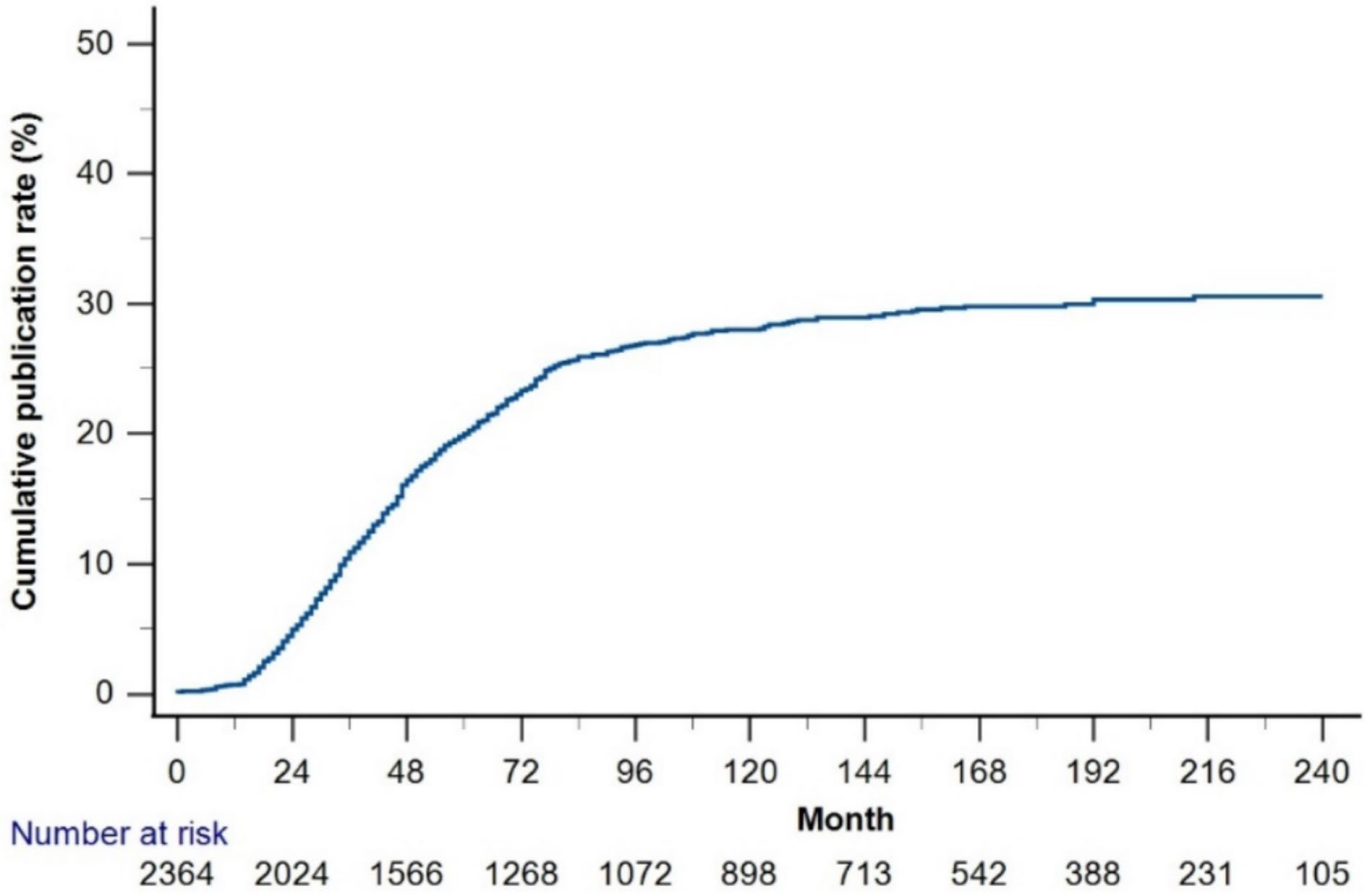
Cumulative publication rate curve since the enrollment of randomized clinical trials.

The cumulative publication rate of all trials was <30% (Figure 4). The 1-, 2-, 3-, 5-, and 7-year cumulative publication rates since enrollment were 0.6, 4.4, 9.5, 16.7, and 20.8%, respectively. Table 3 summarizes the RCT characteristics based on publication status. Compared to the unpublished RCTs, published RCTs were more frequently retrospectively registered (56.6% vs. 49.6%), funded by the industry (49.6% vs. 39.6%), and conducted in North America (30.1% vs. 14.8%) (all *p* < 0.001). The published RCTs included larger sample sizes than the unpublished RCTs (median 124.0 [58, 307] vs. 88 [48, 220]; [samples more than 200 groups] 36.6% vs. 23.7%, *p* < 0.001). Compared to the published RCTs, the unpublished RCTs focused more on medication (83.9% vs. 80.5%, *p* = 0.009), natural medicine and extracts (28% vs. 23.8%, *p* < 0.001), and pharmacokinetics (5.7% vs. 2.0%, *p* = 0.013).

**Table 3:** Characteristics and study design of RCTs according to publication status.

Variables	Published (*N* = 558)	Unpublished (*N* = 1,852)	*p*-value
Sex			0.194
Both female and male subjects	527 (94.4%)	1,713 (92.5%)	
Female subjects	10 (1.8%)	58 (3.1%)	
Male subjects	21 (3.8%)	81 (4.4%)	
Age			0.04
Adults	517 (92.7%)	1,685 (91.0%)	
≤18	19 (3.4%)	48 (2.6%)	
19–44	10 (1.8%)	72 (3.9%)	
45–64	7 (1.3%)	39 (2.1%)	
≥65	5 (0.9%)	8 (0.4%)	
Location			<0.001
Europe	95 (18.0%)	317 (17.1%)	
Asia	130 (24.6%)	892 (48.2%)	
North America	159 (30.1%)	275 (14.8%)	
Oceania	9 (1.7%)	34 (1.8%)	
South America	9 (1.7%)	39 (2.1%)	
Africa	3 (0.6%)	6 (0.3%)	
Multi-continent	124 (23.4%)	289 (15.6%)	
Sample size			
	124 (58.0–307.0)	88 (48.0–220.0)	<0.001
Less than 50	118 (21.1%)	520 (28.1%)	<0.001
50–99	124 (22.2%)	510 (27.5%)	
100–200	104 (18.6%)	336 (18.1%)	
More than 200	204 (36.6%)	438 (23.7%)	
NA	8 (1.4%)	48 (2.6%)	
Type of registration			0.003
Prospective	242 (43.4%)	934 (50.4%)	
Retrospective	316 (56.6%)	918 (49.6%)	
Status			<0.001
Recruiting	24 (4.3%)	273 (14.7%)	
Not recruiting	534 (95.7%)	1,579 (85.3%)	
Masking			<0.001
Blinding	424 (76.0%)	1,118 (60.4%)	
Open label	115 (20.6%)	478 (25.8%)	
NA	19 (3.4%)	256 (13.8%)	
Funder			<0.001
Industry	277 (49.6%)	734 (39.6%)	
Non-industry	278 (49.8%)	1,088 (58.7%)	
NA	3 (0.5%)	30 (1.6%)	
Intervention model			0.516
Parallel assignment	425 (76.2%)	1,471 (79.4%)	
Sequential assignment	7 (1.3%)	23 (1.2%)	
Factorial assignment	12 (2.2%)	30 (1.6%)	
Crossover assignment	89 (15.9%)	263 (14.2%)	
NA	25 (4.5%)	65 (3.5%)	
Type of intervention			0.009
Medication	449 (80.5%)	1,553 (83.9%)	
Management style	25 (4.5%)	57 (3.1%)	
Lifestyle	75 (13.4%)	184 (9.9%)	
Others	9 (1.6%)	58 (3.1%)	
Medication classification			<0.001
Statin	52 (11.6%)	163 (10.5%)	
Cholesterol absorption inhibitor	9 (2.0%)	42 (2.7%)	
Fibrates	3 (0.7%)	59 (3.8%)	
Niacin	2 (0.4%)	25 (1.6%)	
Omega-3 fatty acids	25 (5.6%)	63 (4.1%)	
PCSK9	76 (16.9%)	111 (7.1%)	
Emerging lipid-lowering drugs	44 (9.8%)	169 (10.9%)	
Natural medicine and extracts	107 (23.8%)	435 (28.0%)	
Drug combination	111 (24.7%)	347 (22.3%)	
Others	20 (4.5%)	139 (9.0%)	
Primary outcome			0.013
Efficacy and safety	462 (82.8%)	1,502 (81.1%)	
Pharmacokinetics	11 (2.0%)	106 (5.7%)	
Adherence	6 (1.1%)	11 (0.6%)	
Plaque and CVD	25 (5.2%)	77 (4.2%)	
Endothelial function and inflammation	20 (3.6%)	66 (3.6%)	
Others	30 (5.4%)	90 (4.9%)	

Data are expressed as number (percentage) or median (inter-quartile range).

NA, not available; RCTs, randomized clinical trials.

## 4 Discussion

To the best of our knowledge, this is the first study to comprehensively assess the characteristics of registered dyslipidemia-related RCTs. The number of registered hyperlipidemia-related RCTs increased rapidly since 2005. These RCTs primarily focused on adults, with efficacy and safety as the primary outcomes. The interventions involved medication. Nonetheless, few RCTs focused on age-specific populations, exclusively on women, adherence, and management-style interventions. RCTs involving age-specific participants had small sample sizes and a high proportion of interventions using natural medicine and extracts. The cumulative publication rate of these RCTs was <30% after enrollment.

The majority (91.4%) of the RCTs enrolled adults, with a median sample size of 100 [50, 245]. In contrast, the RCTs that included age-specific participants had smaller sample sizes than those involving adults (median 56 [27, 108] vs.100 [50, 245], *p* = 0.047). Small sample sizes are unreliable for assessing the effect size and may even yield spurious results. Although our analysis identified 125 large-scale RCTs with over 1,000 participants, these RCTs represent only approximately 5% of the total 2,410 RCTs included in our study. While some RCTs had large sample sizes, demographic variable-based stratification often yielded inadequate sample sizes for each subgroup (8). A small sample size has no universal definition and remains controversial. Sample size calculations for clinical trials should be based on robust parameter assumptions, such as the power of a test (*β*) and significance level (*α*) (9), which were neglected in the RCTs on the ICTRP. Therefore, sample size calculations should be comprehensively described during registration. Modern evidence-based medicine, particularly RCTs, encounters challenges when studying older populations, which are often underrepresented in individual trials. This limitation may help explain why only 2.6% of the dyslipidemia-related RCTs on the ICTRP focused on participants aged ≥65 years.

Concerning lipid levels, studies have strongly emphasized the implication of “the earlier, the better” (10). Lipid disorders have been associated with subclinical atherosclerosis and CVD across the lifespan, affecting not only older adults but also children and young adults (2, 11–13). In China, trends in serum lipid levels have worsened among children and adolescents, while favorable trends have been reported in the US (11, 14). Despite these improvements among US adolescents, the incidence of lipid disorders remains high, with rates ranging from 19 to 25% (11). Childhood lipid disorders often persist into adulthood (13); however, only 2.8% of the RCTs involved children and adolescents in the present study. Future research on dyslipidemia must include more diverse populations, with an increased focus on younger demographics, to provide evidence-based foundations for precision prevention strategies.

The impact of dyslipidemia in early life on long-term health extends beyond adolescence and is also applicable to young adults (15). A South Korean study reported that even modest increases in lipid levels were associated with an increased risk of CVD among young adults aged 20–39 years (16). Similar results were reported in the Framingham Offspring Study involving adults aged 40–50 years who did not have CVD at the age of 55. Notably, the association between hyperlipidemia in young adulthood and the risk of CVD remained significant even after adjusting for non-HDL-C levels at the age of 55 (13). In young adults with a low risk of CVD, lowering LDL-C levels effectively reduced the occurrence of major cardiovascular events, showing results comparable to those seen in older adults (17). However, current guidelines recommend quantitative risk assessments using 10-year risk equations starting at age 40, with limited recommendations for apparently healthy young adults aged 20 to 39 years (18). Coincidently, cardiovascular mortality rates in young adults, particularly among women, have plateaued, despite a marked decrease in overall cardiovascular mortality in recent decades (19). Lipid levels in women are more atherogenic and are influenced by transitional life stages, such as the menstrual cycle, pregnancy, breastfeeding, and menopause (20). For example, 38.5% of women with a history of gestational diabetes mellitus develop dyslipidemia in the early postpartum period (21). Despite this, only <1% of the RCTs involved women aged 19–44 years, according to the data on the ICTRP. The next era of CVD prevention should focus on prioritizing trials in younger populations and developing precision medication management (15). There is a critical need for more high-quality RCTs involving younger demographics, particularly women, to address the existing gaps in research.

Lipid-lowering therapies (LLTs), including LDL-C, TG, and lipoprotein(a), effectively reduce major vascular events (18). In this study, more than 80% of dyslipidemia-related RCTs focused on medication interventions and the efficacy and safety of LLTs. However, parallel assignment (78.7%) was the most frequently used intervention model, while only 14.6% of dyslipidemia-related RCTs were designed with a crossover assignment. Statins—first-line drugs for CVD prevention and treatment—accounted for 10.7% of the RCTs. TG-lowering drugs, such as fibrates and omega-3 fatty acids, accounted for 4.4 and 3.1% of RCTs, respectively. Despite the importance of achieving target LDL-C levels and adhering to LLTs, suboptimal outcomes continue to be a concern (22). Guidelines recommend drug combination therapy for patients who are statin-intolerant or do not meet their target LDL-C levels (18). Approximately 22.9% of RCTs focused on drug combination therapy. In addition, emerging lipid-lowering drugs accounted for only 10.6% of RCTs. The development of novel treatment modalities and therapeutic targets holds promise for reducing the risk of adverse cardiovascular events, despite the limited data available (23). Continuous investigation into emerging lipid-lowering drugs is essential to identify novel molecular targets, and researchers should prioritize diverse intervention models (such as parallel-group, crossover, cluster, or other types) in RCT research.

Interestingly, natural medicine and extracts (27.1%) accounted for the largest proportion of medication-related RCTs among adults. This trend was also observed in the age-specific trials. Nutraceuticals that are certified for their lipid-lowering effects have garnered research interest (24). However, the cardiovascular benefits of nutraceuticals have not yet been reported; therefore, they cannot replace traditional lipid-lowering drugs (25). Chinese herbal medicine represents another important category within natural medicine and extracts. Chinese herbal remedies include single herbs, Chinese patent medicines, and compound formulas; all grounded in over 2,000 years of experiential knowledge in treating diseases (26). The effective components in Chinese herbal remedies can delay the formation of atherosclerotic plaque by protecting endothelial cells, inhibiting inflammatory reactions and lipid deposition, regulating gut microbiota, lowering antioxidants, reducing foam cell formation in macrophages, and decreasing lipid peroxidation reactions (27, 28). Multiple active ingredients in Chinese herbs offer cardiovascular benefits and are used in the treatment of CVD (29). However, the limitations of these remedies include single administration methods, poor water solubility, low bioavailability, and weak targeting capabilities (30). The combination of modern scientific technologies, such as nanotechnology, and Chinese herbal medicine provides a more scientific approach to addressing these shortcomings in clinical applications (27, 30). Traditional Chinese medicine—one of the oldest healing systems—encompasses herbal medicine, acupuncture, moxibustion, massage, food therapy, and physical exercise such as Tai Chi (26). Acupuncture—categorized as “other intervention” in the present trials—positively regulates lipid metabolism (31). Novel molecular targets and therapeutic strategies for dyslipidemia and CVD may emerge with advancements in modern scientific techniques, combined with the expanding scope of Chinese herbal medicine research.

Patient non-adherence to LLTs contributes to the failure to achieve LDL-C goals (32). Low adherence to treatment is a major public health problem, adversely affecting morbidity, mortality, and healthcare costs (33). Real-world studies have observed high non-persistence rates and poor adherence across all LLT regimens (32). However, only 1% of the dyslipidemia-related RCTs examined adherence as the primary outcome both in adult and age-specific groups. These data highlight the inadequate attention given to adherence. Multifactorial contributors to poor adherence often involve both patient and physician factors, in addition to therapy-related factors. Common barriers include insufficient knowledge about LLTs, limited implementation strategies, and high costs (32). Management style—a group of interventions—encompasses implementation strategies such as shared decision-making, decision tools, digital tools, physician education programs, and pharmacy-based programs. However, management style-related RCTs accounted for only 3.4, 3.5, and 2.9% of dyslipidemia-related RCTs in the total population, adult group, and age-specific group, respectively. This finding highlights the need for more RCTs focused on management styles and adherence. Researchers should prioritize implementing novel strategies to improve treatment adherence.

In this study, the cumulative publication rate of dyslipidemia-related RCTs after enrollment was <30%. This result is similar to or marginally lower than the findings from other RCTs, such as those on osteoporosis (30.5%) and type 1 diabetes (less than 40%) (34, 35). This discrepancy may be attributed to the inclusion of incomplete RCTs that were still in the recruitment stage. Selective publication bias and discrepancies between expected and actual results have been recognized as key barriers to the dissemination of trial findings (34). Blumenthal et al. (36) found that 20% of researchers withheld results due to financial or reputational concerns.

Investigators have the moral and ethical responsibility to register and publish the results of all clinical trials (37). Publishing clinical trial results provides a reliable basis for evidence-based medicine, facilitates the establishment of health policies, aids clinicians in decision-making, and promotes the development of public health and clinical medicine (38). Failure to publish results undermines the individual contributions of research participants and reduces public trust in clinical science (39). Non-publication of RCTs represents a waste of human and financial resources, leads to biased evidence, and violates the ethical obligation to share results and reduce harm (40). Trial registration increases mandatory transparency in research; however, it remains insufficient to reduce publication bias. Funders, journals, and ethics committees should prioritize enhancing mandatory transparency and avoiding publication bias. To address this issue, we emphasize the need for stricter adherence to prospective registration mandates and the enforcement of results submission on the ICTRP. We further propose the inclusion of mandatory fields in the ICTRP, including “Post-trial completion publication status” and “Reasons for non-publication,” to enhance transparency. Other innovative strategies should also be explored to address low publication rates in the future.

## 5 Study strengths and limitations

The strength of this study is that it presents the first comprehensive overview of registered dyslipidemia-related RCTs. However, there was a notable lack of trials specifically designed for age-specific populations, women, adherence, and management style. Furthermore, researchers should focus on improving publication rates. This study has several limitations. First, the trial data obtained from the ICTRP were often incomplete and outdated, which might have resulted in the omission of protocols or critical information in the analysis. Second, this study provides only an overview of the characteristics of registered dyslipidemia-related RCTs. The advantages and disadvantages of the research could not be investigated owing to insufficient information. Although the ICTRP provides comprehensive coverage through its primary registries, there might be some nuances or additional details that could be obtained by accessing these registries directly. Finally, since the ICTRP data were provided by researchers, they cannot be further verified and validated by the platform.

## 6 Conclusion

In conclusion, to the best of our knowledge, this is the first study to present a comprehensive overview of registered dyslipidemia-related RCTs. High-quality RCTs should prioritize younger and older populations, especially women, and incorporate diverse intervention models. The majority of dyslipidemia-related RCTs lack published results and insufficiently address adherence. To address the issue of low publication rates, it is essential to enforce mandatory trial registration, ensure transparent reporting, and implement innovative strategies.

## Data Availability

The raw data supporting the conclusions of this article will be made available by the authors, without undue reservation.

## Footnotes

^1^ https://www.who.int/clinical-trials-registry-platform
